# Exploring the Link Between Enriched Environment and Depression: Insights From Human Participants

**DOI:** 10.62641/aep.v53i5.1927

**Published:** 2025-10-05

**Authors:** Rodrigo Guiza-Zayas, Martha Ontiveros Uribe, Nadia Palomera-Garfias, Estefania Magaña-Saavedra, Mónica Flores-Ramos

**Affiliations:** ^1^Dirección de Enseñanza, Instituto Nacional de Psiquiatría, 14370 Ciudad de México, Mexico; ^2^Escuela Superior de Medicina, Instituto Politécnico Nacional, 48300 Ciudad de México, Mexico; ^3^Departamento de Servicio Social, Universidad del Valle de México, campus Coyoacán, 04910 Ciudad de México, Mexico; ^4^Laboratorio de Epidemiología Clínica, Subdirección de Investigaciones Clínicas, Instituto Nacional de Psiquiatría Ramón de la Fuente Muñiz, 14370 Ciudad de México, Mexico

**Keywords:** major depressive disorder, environmental enrichment, depressive symptoms, brain plasticity, clinical relevance

## Abstract

**Background:**

Major depressive disorder (MDD) is a prevalent neuropsychiatric condition associated with significant functional impairment and reduced quality of life. Environmental enrichment (EE), a model encompassing cognitive, social, and physical activities, has demonstrated antidepressant effects in animal models through mechanisms involving brain plasticity. In humans, the influence of EE on depressive symptoms and its clinical significance remain under investigation. This study evaluated the relationship between EE domains and the clinical symptoms of MDD, focusing on the possible modulatory effects of EE on depressive symptomatology.

**Methods:**

We conducted an observational, cross-sectional study involving 50 adults diagnosed with MDD. Depressive symptoms were assessed using the 17-items Hamilton Depression Rating Scale (17-HDRS), and EE levels were measured using the Environmental Enrichment Indicator (EEI), which evaluates cognitive, social, and physical activity domains. Correlations between depressive symptoms and EEI domains were analyzed.

**Results:**

Participants with higher 17-HDRS scores generally exhibited lower levels of EE. Additionally, individuals with more severe depressive symptoms were less likely to engage in cognitive activities compared to those with milder symptoms; however, this difference was not statistically significant (Kruskal–Wallis test, H = 3.82, df = 2, *p* = 0.14). Notably, higher EE levels were observed among younger participants.

**Conclusions:**

The level of EE in individuals with depression may affect symptom severity. Further studies in clinical populations are needed to clarify the relationship between EE and depressive symptoms.

## Introduction

Major depressive disorder (MDD) is a prevalent mental health condition and a leading cause of disability worldwide. MDD accounts for the highest number of years lived with disability in 56 developing and low-income countries [1]. MDD is associated with substantial functional impairment, an increased risk of chronic illnesses, and high early mortality, whether due to comorbid conditions or suicide [2]. Treatment for depression typically involves a multidisciplinary approach that addresses both neurobiological mechanisms and psychosocial stressors, integrating pharmacologic and psychotherapeutic interventions.

Depression is marked by a decline in the quality and frequency of social interactions and decreased engagement in cognitive and physical activities, all of which negatively affect its course and prognosis. Depressive symptoms are inversely associated with quality of life, impairing somatic, psychological, cognitive, and social functioning. This relationship has been confirmed in previous research [3]. For instance, a preclinical study reported the role of environmental factors in depression, particularly through research on environmental enrichment (EE) in rats [4]. Rats exposed to stimulus-rich environments exhibited greater resilience [5], a decreased risk of depressive behaviors, and diminished learned helplessness, even when genetically predisposed to depressive behaviors [6]. These antidepressant-like effects of EE highlight the effect of environmental and lifestyle factors on the onset and progression of depressive symptoms [7].

EE refers to a combination of physical, social, motor, somatosensory, and cognitive stimuli [8] and has demonstrated beneficial effects on metabolism, cognitive function, immune response, anxiety, and depression in animal studies [9]. These effects are thought to be mediated by various biological factors, including growth factors, neurotransmitters, and neurotrophins such as brain-derived neurotrophic factor (BDNF) [8, 10, 11, 12]. Core components of EE include cognitive, social, and physical activities [13, 14, 15]. Sequential exposure to both physical and cognitive stimuli has been shown to enhance neuroplasticity [13], although the mechanisms underlying EE’s effects remain incompletely understood [16, 17].

Depression is associated with low EE because cognitive deficits among individuals with depression can worsen the course of the disorder and impair overall functioning [18, 19, 20]. In addition, the quality and frequency of social interactions play a vital role in evaluating and coping with stress, serving as buffers against its negative effects [21, 22]. Physical activity has also been identified as a predictive factor for preventing the onset of new depressive episodes [23] and has demonstrated therapeutic benefits across varying levels of symptom severity [24].

Despite increasing evidence supporting the benefits of EE, its application in human populations remains limited. Measuring individuals’ daily activities may serve as a proxy for assessing the level of EE in their lives. To address this, our research group developed and validated the Environmental Enrichment Indicator (EEI). The EEI has been standardized in Spanish for use in the Mexican population and captures cognitive challenges encountered in daily life, the frequency and quality of social interactions, and the regularity and intensity of physical activity [25]. Preliminary findings using this tool revealed a relationship between cognitive and social activities and serum levels of BDNF in individuals with depression, suggesting that the EEI may reflect molecular-level modulation of depressive symptoms [26]. Based on these findings, we examined the association between EEI scores and the clinical symptoms of individuals with depression.

## Methods

Participants were recruited from inpatient units, continuous psychiatric care programs, pre-consultation services, and outpatient clinics at the National Institute of Psychiatry in Mexico City. All participants met the *Diagnostic and Statistical Manual of Mental Disorders, Fifth Edition* (*DSM-5*) criteria for MDD. Each potential participant received a detailed explanation of the study procedures, and those who agreed to participate were read the informed consent form and asked to sign it. Evaluations were conducted between March 2022 and February 2023 by a specialist psychiatrist who administered both the 17-item Hamilton Depression Rating Scale (17-HDRS), and the EEI to each participant. Inclusion criteria: Adults aged 18 to 60 years who could read and understand the questionnaires, met *DSM-5* criteria for MDD, and scored 13 or higher on the 17-HDRS.

Exclusion criteria: Individuals were excluded if they had used antidepressants, anxiolytics, or mood stabilizers within the past 8 weeks; had received electroconvulsive therapy or transcranial magnetic stimulation in the previous year; or had depression secondary to a medical condition. Additional exclusion criteria included chronic medical illness, uncontrolled glycemic levels, uncontrolled thyroid disease, suicidal ideation, psychotic symptoms at the time of assessment, a history of manic or hypomanic episodes, or a moderate to severe substance use disorder as defined by the *DSM-5*.

### Evaluations

#### Sociodemographic and Clinical Variables

Demographic characteristics, including sex, age, educational background, marital status, and occupational status, were collected through in-person interviews by using a structured questionnaire designed for this study.

#### Hamilton Depression Rating Scale

Depressive symptoms were assessed using the 17-HDRS, which is widely regarded as the gold standard for evaluating the severity of depressive symptoms. The scale includes 17 items rated on either a 0–4 scale (0 = symptom absent; 4 = symptom severe) or a 0–2 scale (0 = absent; 1 = slight or trivial; 2 = clearly present). Total scores range from 0 to 54. Generally, scores of 0–7 indicate remission or no depression; 8–13 suggest mild depression; 14–18 indicate moderate depression; 19–22 reflect severe depression; and scores of 23 or higher indicate very severe depression [27]. The validated Spanish version of the HDRS was used in this study [28].

#### Environmental Enrichment Indicator 

The EEI is a self-administered, validated questionnaire designed to assess EE levels across three domains: social activities, cognitive activities, and physical activity. Based on scores within each domain, overall EE is categorized as low, moderate, or high [25].

• Social activities: This domain includes 22 items assessing the frequency of social activities over the past month, using a 5-point Likert scale (0 = did not perform the activity; 4 = performed it daily). An additional eight items evaluate satisfaction with social engagement (0 = very unsatisfied; 4 = very satisfied). Frequency and satisfaction scores are calculated separately and then summed to determine overall social integration. Social activity levels are classified as low (0–78), moderate (79–98), or high (99 and above).

• Cognitive activities: This domain comprises 25 items evaluating the frequency of cognitive activities over the past month using a 5-point Likert scale (0 = did not perform the activity; 4 = performed it daily). The total score is the sum of all items, with higher scores indicating greater cognitive engagement. Scores are categorized as low (0–42), moderate (43–56), or high (57 and above).

• Physical activity: This domain includes seven items measuring the frequency (days per week), duration (minutes per day, except sedentary time measured in hours per day), and intensity (sedentary, walking, moderate, or vigorous) of physical activity. Scores are calculated based on energy expenditure in metabolic equivalents of task (METs), with classifications as follows: low (0–600 METs), moderate (601–1499 METs), and high (1500+ METs).

When considering all domains, we used the following classification:

Low EE: Two or three domain (cognitive, social, and physical dimensions) fall in the low category.

Moderate EE: Two or more domains fall into the moderate category, or one domain is low, one moderate, and one high.

High EE: Two or more domains fall into the high category.

#### Assessment Procedure

Participants underwent a 30-minute interview during which clinicians collected clinical and sociodemographic information and administered the 17-item HDRS. Then, participants completed the EEI by responding to items assessing their engagement in daily cognitive activities, social interactions, and the frequency and intensity of physical activity, as outlined by the scale.

#### Ethical Considerations

The study was approved by the ethics and research committees of the National Institute of Psychiatry under protocol number CEI/C/010/2022. All procedures were carried out in adherence to the Declaration of Helsinki.

### Statistical Analysis 

Descriptive statistics, including measures of central tendency and dispersion, were used to summarize the data. Bivariate correlations were performed, and a post hoc Bonferroni test was applied. Analysis of variance (ANOVA) was used to compare 17-HDRS scores across the three levels of EEI (low, moderate, or high) and across the three levels of each individual domain. When the assumption of homogeneity of variances was violated, the Kruskal–Wallis test was used instead. ANOVA was also applied to compare age across the three EEI levels. The Kolmogorov-Smirnov test was used to assess data distribution, and the Levene test was used to examine the homogeneity of variances. All statistical analyses were conducted using IBM SPSS Statistics for Windows, Version 20.0 (Armonk, NY, USA: IBM). A *p* value of ≤0.05 was considered statistically significant.

## Results

A total of 50 adults diagnosed with MDD were evaluated. The sample included 84% of women and 16% of men. The mean age was 33.42 years. Additional demographic characteristics are presented in Table 1.

**Table 1.** **Sociodemographic characteristics**.

Characteristic		n (%) or mean (s.d)
Sex		n (%)
	Men	8 (16%)
	Women	42 (84%)
Age		mean (s.d)
		33.42 (11.23)
Job Status		n (%)
	Employee	20 (40%)
	Unemployed	9 (18%)
	Student	16 (32%)
	Housewife	5 (10%)
Marital Status		n (%)
	Married	11 (22%)
	Single	34 (68%)
	Divorced	5 (10%)
Education		n (%)
	Primary School	3 (6%)
	Middle school	6 (12%)
	High school	20 (40%)
	Bachelor’s degree	16 (32%)
	Postgraduate	5 (10%)

We analyzed mean scores across the three EEI domains for the full sample of 50 participants. In the physical activity domain, measured in total METs, the mean score was 1184.96 (SD = 1833.60). The cognitive activity domain showed a mean score of 50.26 (SD = 17.66), whereas the social activity domain had a mean score of 32.00 (SD = 11.08). These results provide a general overview of the distribution of EE activities among participants.

**Fig. 1.**
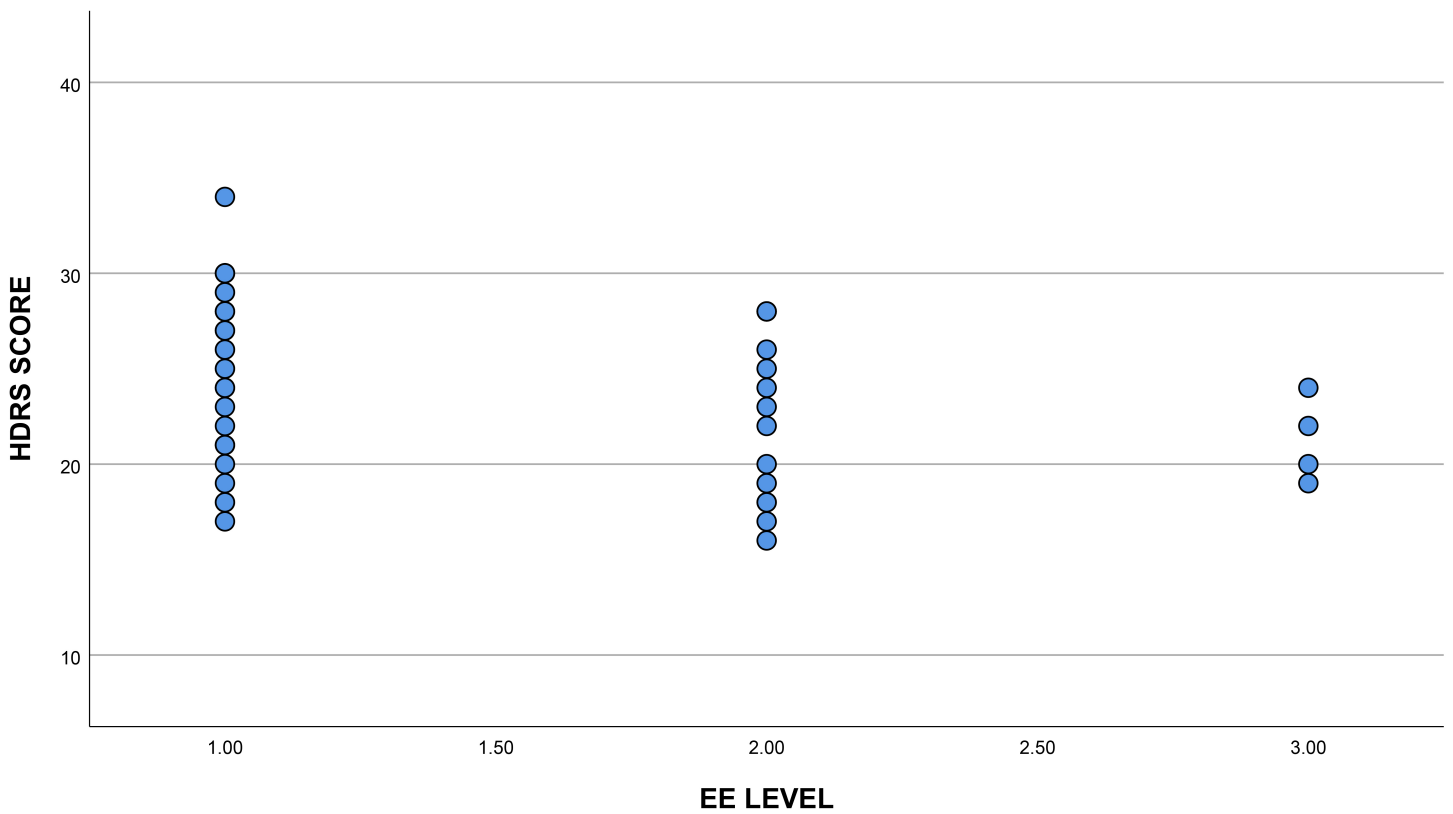
**Relationship between EE levels and HDRS**. Fig. 1 illustrates the relationship between HDRS scores and the EE level in patients with depression. Patients with more depressive symptoms were more likely to fall into the lower EE category. HDRS, Hamilton Depression Rating Scale; EE, Environmental Enrichment. 1: Low EE level, 2: Medium EE level, 3: High EE level.

**Fig. 2.**
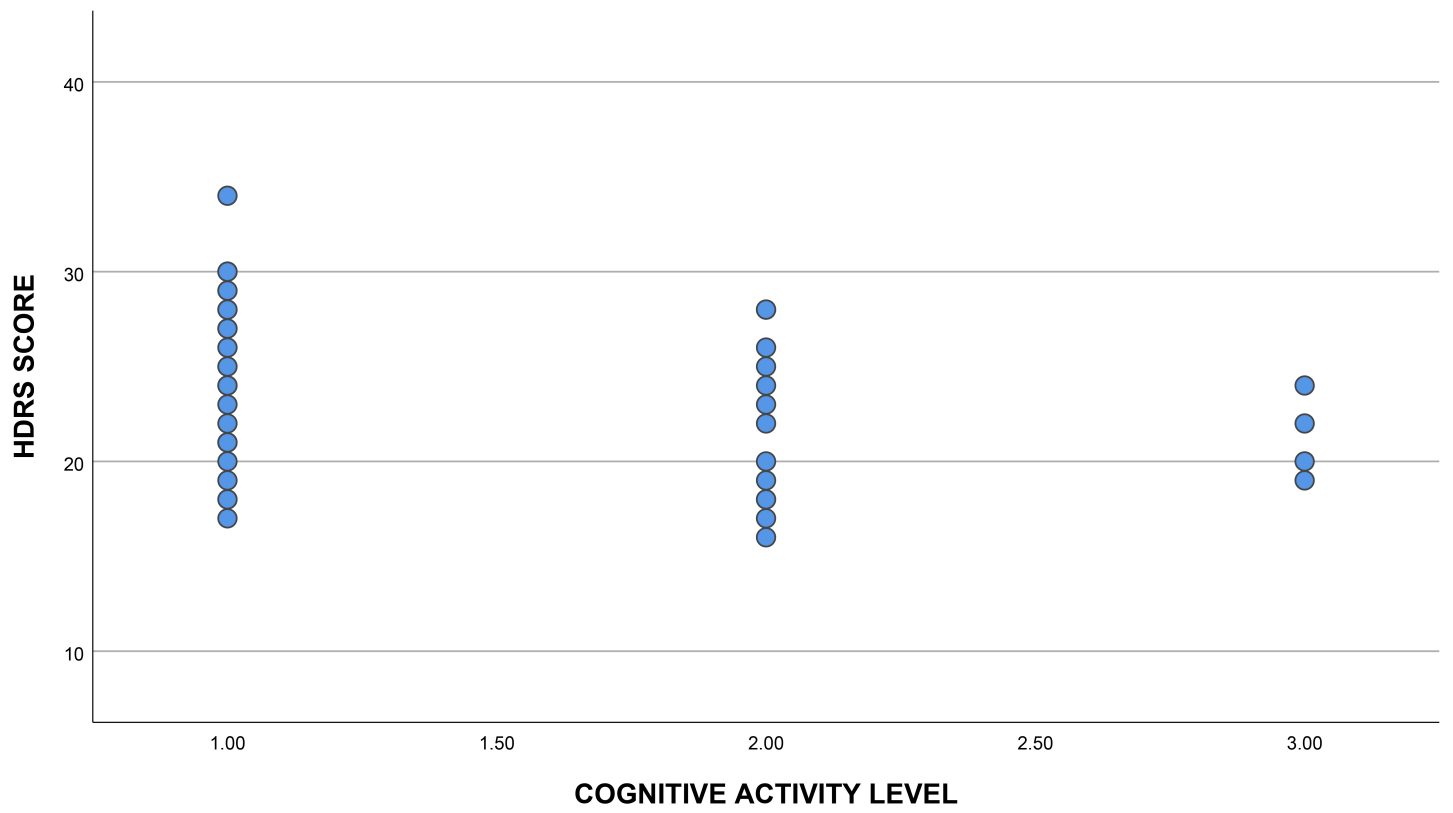
**Relationship between levels of depressive symptoms and participation in cognitive activities**. Fig. 2 illustrates the relationship between HDRS scores and cognitive activity levels. Patients with higher depressive symptoms had lower cognitive activity levels. HDRS, Hamilton Depression Rating Scale. 1: Low cognitive activities level, 2: Medium cognitive activities level, 3: High cognitive activities level.

We compared 17-HDRS scores across participants classified as having low, moderate, or high EE and found that those with low EE had higher levels of depressive symptoms (ANOVA, F = 3.00, *p* = 0.05; Fig. 1), those results showed a trend but were not statistically significant. In addition, participants with higher HDRS scores tended to engage in fewer cognitive activities, with a mean HDRS score of 24.95 in the low cognitive activity group versus 22.50 in the high cognitive activity group. However, this difference was not statistically significant (Kruskal–Wallis test, H = 3.82, df = 2, *p* = 0.14; Fig. 2). Lastly, while participants in the high EE group were younger on average than those in the other groups, the age difference was not statistically significant (Table 2).

**Table 2.** **Age and depressive symptoms according to EE level**.

		Descriptives				
		N	Mean	s.d	F	*p*
	EE level					
HDRS	Low	32	25.06	2.21	3.00	0.05
	Moderate	14	22.29	4.32		
	High	4	21.25	2.21		
Age	Low	32	35.28	11.30	1.31	0.28
	Moderate	14	30.64	10.24		
	High	4	28.25	13.33		
Total		50	33.42	11.23		

EE, Environmental enrichment; s.d, Standard deviation; HDRS, Hamilton Depression Rating Scale.

Correlation analysis between EEI domains and depressive symptoms revealed a significant negative correlation between guilt symptoms and the social activity domain (r = –0.30, *p* = 0.03). A significant negative correlation was also found between early insomnia and physical (r = –0.30, *p* = 0.04), and cognitive activities (r = –0.29, *p *= 0.04). Similarly, a significant negative correlation was observed between middle insomnia and cognitive activities (r = –0.41, *p* = 0.00; Table 3). Lastly, a significant negative correlation was found between insight symptoms and engagement in cognitive activities (r = –0.33, *p* = 0.02; Table 3). After applying Bonferroni correction for multiple correlations, none of the correlations between depressed symptoms and EEI domains remained statistically significant.

**Table 3.** **Correlation between environmental enrichment domains and depressive symptoms**.

	Physical activity		Cognitive activity		Social activity	
	r	*p*	r	*p*	r	*p*
Depressive mood	0.17	0.24	0.03	0.85	–0.02	0.90
Feelings of guilt	0.00	1.00	0.01	0.94	–0.30*	0.03
Suicide	–0.10	0.48	–0.09	0.56	0.02	0.87
Early insomnia	–0.30*	0.04	–0.29*	0.04	–0.03	0.84
Middle insomnia	–0.16	0.28	–0.41**	0.00	–0.06	0.65
Late insomnia	0.11	0.46	–0.14	0.34	–0.09	0.52
Work and activities	0.01	0.92	0.25	0.08	0.05	0.72
Psychomotor inhibition	–0.15	0.32	–0.11	0.47	0.24	0.09
Psychomotor agitation	0.12	0.42	–0.10	0.51	0.01	0.94
Psychic anxiety	–0.08	0.57	–0.05	0.75	–0.09	0.49
Somatic anxiety	–0.23	0.11	–0.18	0.20	0.19	0.19
G.I somatic symptoms	0.15	0.29	–0.14	0.34	–0.28*	0.05
Somatic symptoms	–0.09	0.54	0.04	0.76	0.22	0.13
Genital Symptoms	0.16	0.26	0.18	0.22	–0.07	0.65
Hypochondria	–0.04	0.80	–0.06	0.70	0.03	0.84
Weight loss	0.09	0.53	0.21	0.14	0.19	0.18
Insight	0.04	0.81	–0.33*	0.02	–0.18	0.22

* significant at *p* ≤ 0.05; ** significant at *p* ≤ 0.01.

## Discussion

EE has been extensively studied in animal models; however, translating this concept to humans presents considerable challenges due to the limited ability to manipulate human environments [29]. In our study, we approximated EE by measuring individuals’ daily activities and the extent of their engagement in enriching experiences. However, the instrument used did not allow for assessment of the spatial complexity of participants’ environments, as recommended by other researchers [29]. A novel clinical approach to evaluating EE involves assessing the complexity of housing and lifestyle factors [30]. For example, Khalil and colleagues reported an inverse relationship between complex housing—characterized by physical activity and novelty—and symptoms of depression, anxiety, and impaired cognitive function, all of which are associated with hippocampal neurogenesis.

Despite this limitation, our scale offers a reasonable approximation of participants’ environments, as it incorporates the key components of EE: cognitive, social, and physical activities. Additionally, the scale evaluates participants’ satisfaction with these activities, which is an important consideration in human studies [31].

Our findings indicate that patients with higher levels of depressive symptoms exhibited lower levels of EE. This result aligns with evidence from animal models, where exposure to novel and enriched environments has been shown to reduce depressive-like behaviors [32, 33] and may prevent abnormal behaviors in models of psychiatric disorders [34, 35]. Notably, in our sample, a substantial proportion of patients (64%) had low EE levels, while only four participants (8%) exhibited high EE levels. However, given the cross-sectional nature of the study, causality cannot be inferred. A bidirectional relationship is a more plausible explanation—patients with depression may be less inclined to engage in cognitive, social, and physical activities, resulting in lower EE.

When examining specific components of EE, cognitive activities appeared to be the most affected. Patients with higher depression scores tended to report lower cognitive engagement, although this difference was not statistically significant. Previous research has shown that individuals with depression often demonstrate impaired cognitive performance [36], likely related to disrupted neurogenesis mediated by BDNF. Furthermore, the link between cognitive deficits and anhedonia—a core symptom of depression—has been previously reported [37, 38]. One possible explanation is that patients with severe depression and pronounced anhedonia lack interest in enriching activities across all domains. Alternatively, low EE levels in such patients may reflect underlying neurobiological changes, such as decreased BDNF levels [26], that contribute to diminished cognitive functioning.

In our sample, younger participants tended to exhibit higher levels of EE, suggesting that younger individuals with depression may be more inclined to engage in enriching activities compared to older counterparts. Notably, the beneficial effects of EE have been shown to be more pronounced during early development of rats [6], and early-life exposure to EE can have lasting effects into adulthood [39]. Cognitive performance may be influenced by EE levels, but it could also reflect age-related neurobiological processes such as neurogenesis. Therefore, it is concerning that older patients in our sample were less engaged in enriched activities, given that EE has been proposed as a protective factor against some of the cellular and behavioral declines associated with aging [39, 40]. However, we cannot rule out the possibility that higher EE levels observed in younger participants are not simply age-related but may also reflect lower HDRS scores. Our data do not allow for a definitive conclusion, as the high EE category included more young participants.

All participants, regardless of depression severity, exhibited low levels of social activity. In animal models, social isolation has been linked to increased depressive-like behaviors [41] and decreased exploration of novel objects [42], the latter of which may reflect increased fear or anxiety.

Although we did not find a statistically significant relationship between depressive symptoms and EE domains, we observed a tendency for reduced engagement in cognitive activities among participants experiencing insomnia. This finding warrants further investigation, as insomnia has been linked to attentional deficits [43]. Evidence suggests that individuals with insomnia perform more poorly on tasks involving attention and episodic memory. Mild but clinically meaningful cognitive impairments are more frequently reported in individuals with insomnia, with deficits in memory and attention often reflecting subtle dysfunction in cognitive inhibitory processes [44]. Additionally, these individuals may experience mild to moderate difficulties in attentional domains, such as choice reaction time, information processing, and selective attention [45].

A key limitation of this study is its cross-sectional design, which precludes conclusions about the directionality of observed associations. It remains unclear whether higher EE levels mitigate depression, or whether patients with severe depression are simply less motivated to engage in enriching activities—potentially due to anhedonia. Furthermore, the absence of a non-depressed control group limits our ability to evaluate the relative importance of social, cognitive, and physical activity in determining EE levels.

Despite the aforementioned limitations, we believe that examining the relationship between enriched environment and depression in humans will allow for the evaluation of the multiple benefits reported in animal models. Our results are an initial approximation to this important field of study.

## Conclusions

Our findings suggest that evaluating the degree of EE in patients with depression is clinically relevant because patients with more severe depressive symptoms exhibited lower EE levels. Future research should focus on monitoring changes in EE domains throughout the course of treatment for MDD and examining how these changes correspond to improvements in depressive symptomatology.

## Availability of Data and Materials

Availability of Data and Ethics Approval is available as requested. Informed consent to participate is not applicable to availability due to ethical committee request.
